# Neonatal Deaths in Rural Southern Tanzania: Care-Seeking and Causes of Death

**DOI:** 10.5402/2012/953401

**Published:** 2012-01-24

**Authors:** Mwifadhi Mrisho, David Schellenberg, Fatuma Manzi, Marcel Tanner, Hassan Mshinda, Kizito Shirima, Beverly Msambichaka, Salim Abdulla, Joanna Armstrong Schellenberg

**Affiliations:** ^1^Ifakara Health Institute, Plot 463 Kiko Ave., Mikocheni Dar es Salaam, Tanzania; ^2^London School of Hygiene & Tropical Medicine, London, WC1E 7HT, UK; ^3^Swiss Tropical and Public Health Institute, 4002 Basel, Switzerland; ^4^Universität Basel, Petersplatz 1, CH-4003 Basel, Switzerland

## Abstract

*Introduction.* We report cause of death and care-seeking prior to death in neonates based on interviews with relatives using a Verbal Autopsy questionnaire. *Materials and Methods. *We identified neonatal deaths between 2004 and 2007 through a large household survey in 2007 in five rural districts of southern Tanzania. *Results. *Of the 300 reported deaths that were sampled, the Verbal Autopsy (VA) interview suggested that 11 were 28 days or older at death and 65 were stillbirths. Data was missing for 5 of the reported deaths. Of the remaining 219 confirmed neonatal deaths, the most common causes were prematurity (33%), birth asphyxia (22%) and infections (10%). Amongst the deaths, 41% (90/219) were on the first day and a further 20% (43/219) on day 2 and 3. The quantitative results matched the qualitative findings. The majority of births were at home and attended by unskilled assistants. *Conclusion.* Caregivers of neonates born in health facility were more likely to seek care for problems than caregivers of neonates born at home. Efforts to increase awareness of the importance of early care-seeking for a premature or sick neonate are likely to be important for improving neonatal health.

## 1. Background

The Millennium Development Goal 4 aims at reducing child mortality by two-thirds by the year 2015. There are an estimated 3.6 million neonatal deaths around the world each year [1, 2]. Although simple, low-cost tools and strategies could prevent many of these deaths, the neonatal period now accounts for 41% of under-five deaths [1, 3]. Besides looking at overall child mortality and infant mortality in the first year of life, neonatal deaths are of great importance and causes of neonatal deaths and care-seeking for the neonates have been neglected in the past. The distribution of causes of neonatal deaths varies substantially between and within countries [1, 3]. About two-thirds of neonatal deaths occur in the African and southeast-asian regions. Although south-asian countries have the largest absolute number of deaths, Sub-Saharan Africa generally has higher rates of neonatal mortality. Less than 3% of neonatal deaths take place in countries with reliable vital registration data for cause of death analysis [3]. Although most studies estimate only the cause of death among neonates, for health programming, it is equally important to understand the care-seeking processes and treatment actions that occurred before each death [4, 5]. A functioning continuum of care between home and hospital is required to minimize potential delays and effectively link women and newborns with care [6].

 In southern Tanzania, births and deaths go largely unregistered and a majority of deaths occur out of reach of the health services [7]. Verbal autopsy (VA) involves structured interviews with bereaved relatives with the aim of estimating a community's mortality experience [8]. An understanding of the interactions of social, behavioural, biological, economic and, environmental characteristics of families and how they relate to causes of death are best studied using a multidisciplinary approach [9–11].

In Tanzania, the neonatal mortality rate was 32 per 1000 live births in 2000–2004 [12]. The 2010 Tanzania Demographic and Health Survey (TDHS) had shown a slight decrease in neonatal mortality [13]. However, there are within-country variations, and neonatal mortality rate was estimated at 43 per 1000 live births in the southern regions of Lindi and Mtwara in 2001–2004 [14]. Information on causes of deaths is important for rational public health planning [15], but this kind of information is rarely available in Tanzania as most deaths are unregistered [3, 11]. The verbal autopsy (VA) technique offers a practical, if imperfect, solution: trained field staff interviews the closest caregiver of the deceased and supplement this with any clinic or hospital records that are available [8, 11, 16–20]. We report an analysis of data collected with VA questionnaires for a sample of deaths reported to have been neonatal in five districts of Lindi and Mtwara regions, southern Tanzania, in order to describe care-seeking prior to death and the main causes of death. 

## 2. Materials and Methods

### 2.1. Study Area

The study was conducted in five districts of Lindi and Mtwara regions in southern Tanzania, a study area that has been described in detail elsewhere [14, 21]. The surveyed districts were Lindi rural, Ruangwa, and Nachingwea (Lindi region); Newala and Tandahimba (Mtwara region). In brief, these areas have a total population of about 900,000 people [12, 22]. There are two main rainy seasons, November to December and February to May. The area has a wide mix of ethnic groups, the most common being Makonde, Mwera, and Yao. These groups frequently intermarry and are predominantly Muslim. Health services are primarily delivered by the public health system. This consists of a network of dispensaries, health centers, and hospitals that offer varying quality of care. There are a few private not-for-profit dispensaries and hospitals run by Christian mission organisations. Three-quarters of the population live within about 5 km of their nearest facility [14]. About one-third of women aged 15–49 years in Lindi and Mtwara are reported to have experienced the loss of at least one child [14].

### 2.2. Methodology

This study was linked to an effectiveness study of intermittent preventive treatment of infants (IPTi) against malaria (http://www.ipti-malaria.org/) [14, 23]. A large household survey carried out from June to October 2007 included all 243,000 consenting households in the area: visited households were georeferenced [24]. A birth history module relating to all live births in the previous 5 years was completed for all consenting women aged 13–49 years. Any woman whose child had died was asked for her consent to participate in a cause-specific mortality survey that followed the main survey (August–November 2009). A representative sample of 300 deaths that had reportedly occurred in children aged 0–28 days between 2004 and 2007 was drawn for the current study using simple random sampling. Verbal autopsies were administered by one of a team of four interviewers between August and November 2007: interviewers had been trained for two weeks by an experienced VA coder. All interviewers worked in all districts. The training included interview technique and probing for dates using local event calendars. A detailed and validated questionnaire adapted from the INDEPTH-Network (http://www.indepth-network.org/) was used and piloted during the training sessions. At the end of the training period, a pilot survey was carried out.

The questionnaire included both open and closed questions and started with the respondent's verbatim account of the circumstances leading to the death of the neonate. The interviewer kept on prompting until the respondent replied that there was nothing else to add. Immediate caregivers (most often mothers, fathers, and grandmothers) were the primary respondents. Dates of births and deaths were ascertained with the aid of information available in any health records available at the household and a local events calendar.

All questionnaires were photocopied before sending to the coders. The main cause of death was coded as described in previous studies [8, 11, 19, 20]. Two physicians independently reviewed the VA forms, and a diagnosis was established as the “probable cause of death” if the two of them agreed. Where this was not the case, a third physician, not knowing anything about the outcome of the first two assessments, provided a further independent assessment. If two of the three agreed, their diagnosis was taken as the “probable cause of death.” If there was no agreement, the case was declared unresolved. When little or no information was available to enable an assignment of cause of death, the coders could agree on the diagnosis being “unknown.” Diagnosis was made according to the International Classification of Diseases, 10th revision (ICD-10, 1990) [25]. Neonatal death was defined as death of a live-born child occurring within 0–28 days of life. The mother or care-giver was asked if she/he sought care outside home while the baby had illness.

### 2.3. Data Processing and Analytical Methods

Analysis of quantitative data was done in Stata (version 10, College Station, TX, USA). Proportions were compared using the chi-square test. For qualitative data analysis, transcripts were typed in Microsoft word and imported to NVivo version 8 for analysis. We applied qualitative content analysis, identifying major key themes from the coded transcripts.

### 2.4. Ethical Approval

The study was undertaken within the framework of the assessment of the community effectiveness of IPTi, a study that was part of the IPTi Consortium [26]. We received ethical approval from the local and national institutional review boards (Ifakara Health Institute, Ifakara, and the Tanzania National Medical Research Coordinating Committee) through the Tanzania Commission for Science and Technology. In addition, ethical and research clearance was also obtained from institutional review board of the London School of Hygiene and Tropical Medicine, UK, and Ethics Commission of the Cantons of Basel-Stadt and Basel-Land, Switzerland. In the household survey, written consent of all household heads was sought and verbal consent from other interviewees.

## 3. Results

### 3.1. Causes and Timing of Neonatal Death

**Figure 1:**
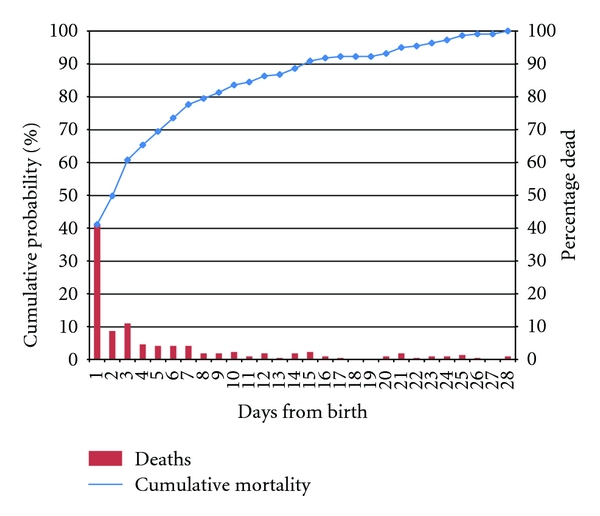
Age at death for neonatal deaths in Lindi and Mtwara.

The main respondents for this study were mothers (50%), fathers (30%), and other relatives (20%). Thirty percent of the respondents had no formal education, 68% had completed primary school, and only 2% completed secondary school. The mean age of the respondents was 33 years old. Of the 300 randomly selected deaths identified and reported as neonatal in the main household survey, the VAs suggested that 11 were older than 28 days at death, 65 were stillbirths and missing data for 5 neonates. Of the remaining 219 neonatal deaths, a diagnosis was agreed for 78% (171/219) causes of death. The most common causes were prematurity (33%), birth asphyxia (22%), infections (10%), and congenital abnormalities (5%) (Table 1). Amongst the deaths, 41% (90/219) were on the first day, a further 20% (43/219) on days 2 and 3, and 39% (86/219) between days 4 to 28 (Figure 1). 

### 3.2. Place of Birth

Among the sample of neonatal deaths, most had been born at home, (63%, 125/199) and were mainly assisted by Traditional Birth Attendants (TBAs) and relatives. Care-seeking in final illness that led to death was more common among children born at a health facility (80% 59/74 versus 24% 30/125) (*P* = 0.0001) (Table 1).

### 3.3. Place of Death

The majority of deaths (69%, 147/214) occurred at home, and only 31% (67/214) occurred at health facility level (Table 1). There was no evidence of differences between care-seeking for boys (43% (47/110); 95% C.I.29–57%) and girls (53% (55/103); 95% C.I. 40–66%) nor between twins and singletons (*P* = 0.09) (Table 1). The vast majority of neonates who died at home (73%, 107/147) had not sought care during the neonatal period.

### 3.4. Perceived Reasons for Neonatal Death

Analysis of qualitative information from VAs revealed four partly interrelated key themes explaining neonatal deaths (i) assistance during labour and delivery, (ii) feeding practices, (iii) access to care and care-seeking and (iv) premature birth (Table 2).

#### 3.4.1. Problems Related to Assistance during Labour and Delivery

Neonates who were delivered at home were assisted by unskilled attendants who cannot handle complications, and sometimes mothers were left unattended for hours. In some extreme cases, mothers were left alone during delivery and experienced prolonged labor, which could potentially risk the life of both mother and neonate. Capacity for emergency obstetric care at health facilities was also lacking. The following statements illustrate these different problems related to assistance during delivery.

*“This was my second pregnancy and it lasted for 9 months. I gave birth safely but I was alone and it was at night. I planned to take the baby to hospital early in the morning but he was no more” *(Mother; 39 years old).

*“This was my third pregnancy and I used to attend clinic at MC dispensary…. I got labour pain and went to MC dispensary to deliver. But one leg of the child got stuck and it took long time to pull it out. After a while, they succeeded to pull that leg out. Moreover, it also became difficult to pull out the baby's head as it was left behind. It took long time for the midwife and the health worker to pull out the baby. Unfortunately, the baby died on her way out…” *(Mother; 30 years old).

#### 3.4.2. Feeding Practices

Breast feeding which commences soon after delivery is most beneficial for the baby and the mother. Although the majority of neonates were breastfed, a few neonates were hardly breastfed in the first three days of life. The most common foodstuffs that were reported to be given to the babies were coconut juice mixed with sugar, tinned milk, cow's milk, or water mixed with sugar and salt. The following statement is typical.

*“My baby was three days old … at around 9 pm, my baby started to cry. I woke up, boiled water and mixed it with sugar and gave it to the baby. The baby was able to breastfeed but I had no milk in my breast. After that event, the baby kept silent and went to sleep. In the middle of the night, I found that my baby had died. I did not see any danger signs before he died” *(Mother; 32 years old).

#### 3.4.3. Access to Care and Care-Seeking Practices

The most common reasons mentioned for a delay in care-seeking for the neonate outside the home include lack of money, the time taken to go to the health facility associated with cash mobilization, distance to the health facility, lack of signs and symptoms of the illness, sudden death, and illness associated with spirits. In addition, it was reported that sick neonates are sometimes taken first to a traditional healer following advice from elders. One mother who had not attended clinic during pregnancy was reportedly denied care as it was her first time to present to the health facility.

*“This was my wife's fifth pregnancy. She was in her nine month of pregnancy. The child's weight was normal and (he) cried immediately after birth. The baby was born with disability in his right leg and could not even turn the leg. This condition lasted for twenty days. We took the baby to NN dispensary but we were referred to the regional hospital … However, we were not able to go due to lack of money to pay for transport. The baby's knee started to swell in the eighteenth day of our baby's life. He eventually died on his 20th day of life” *(Father: 34 years old).

*“This was her first pregnancy. She had no problem at the beginning. When she was eight months pregnant, she experienced labour pains and gave birth safely at 4am at a local dispensary. The child did not cry, and had yellow eyes. On day three, the health worker advised us to go to the hospital. But when we were coming back home to get prepared, the child passed away” *(Grandmother; 60 years old).

* “This was her third pregnancy. In her first pregnancy, she had a stillbirth. In the second pregnancy, she delivered safely and the child is still alive. She had no problems during her last pregnancy. She gave birth under the supervision of my grandmother. Soon after delivery, the child had a fever; with high body temperature and was crying. The baby was taken to the local dispensary but the nurses refused to examine the baby, because her mother did not attend clinic during pregnancy. My wife went back home with the baby but she passed away on the second day”* (Father, 30 years old).

*“This was my first pregnancy. I attended clinic at the district hospital where I got two tetanus injections. I was fine during the whole time of my pregnancy. I experienced labour pain and delivered safely with the assistance from my mother. Soon after birth, the baby cried, breastfed well and breathed well. After two weeks the color of the baby's eye changed to yellow. He was crying all the time and the whole body changed to yellow. The baby's body was hard like a dry fish and he lost energy. He was taken to the district hospital and was administered. The baby was examined so as to identify the causes of the problem but nothing was found. Based on our neighbor's advice, we asked for discharge so as to take our baby to the traditional healer. The kind of illness that our baby had could not be cured in the hospital as it was associated with spirits. The traditional healer told us that we were late but gave us some herbs to swallow and for mixing with water before washing the baby. We went back home but the baby's condition did not change. He cried the whole night and we tried to give him some medicine but it did not help. In the morning of day 3 after the discharge from the hospital, the baby passed away.” *(Mother: 16 years old).

#### 3.4.4. Premature Birth

Premature babies are generally at greater risk for short- and long-term complications. Since the majority of births were at home and assisted by unskilled attendants (TBAs and relatives), there was no mechanism to look after babies born premature. Lack of knowledge on how to handle neonates born premature was common. Twin neonates were more commonly reported to be born premature and experienced more deaths compared to singletons. Lack of breast feeding of the premature babies was reported by mothers as a contributing factor for neonatal death. These findings corroborate the result of the quantitative work that majority of premature birth dies at home as compared to other neonates dying from other causes.

* “This was my first pregnancy. I attended clinic during this pregnancy and got two injections to prevent tetanus. During this pregnancy, I had no health problems. When I was in my seventh month of pregnancy, I experienced labour pain and delivered at home. I was assisted by a traditional birth attendant. My child cried and I breastfed him as normal. On day four, the child had fever and difficult breathing. The baby was sent to the hospital where she was admitted and treated for three days. Afterwards, the baby was discharged and went back home. Generally, the baby was still very weak and died the following day”* (Mother; 20 years old).

*“She was six months pregnant. She used to attend clinic at a local dispensary. She had no health problems during the first trimester. But in her second trimester, she had normal labour pains which lasted a short while and she gave birth to a baby girl. She delivered at home with the help of her neighbors. This was her second child. During the first pregnancy, she had a similar experience but gave birth to a dead baby. The second child was very small but cried and breastfed well soon after birth. However, the child died suddenly at night on the third day” *(Grandmother; 45 years old).

*“This was my fifth child that was born alive. I have lost all babies when they were still very young. My pregnancy lasted for seven months. The baby was very small and could not be breastfed. He only survived for about 14 hours and passed away” *(Mother; 25 years old).

*“This was her third pregnancy and it was in her six month of gestation. After giving birth, the child did not cry properly and was not able to breastfeed for three days. On the third day, the child started to lose energy and closed his eyes; and finally lost his life” *(Father; 29 years old).

*“My wife was in her seven month of pregnancy. One day she started feeling labour pain from around 6pm to 8 pm. She gave birth on the way to the health facility to a baby who was smaller than average. On the second day I went to MN village to inform her parents. When I came back with my mother in law, the baby was no more. The baby couldn't manage to suck on her own as there was no milk. We just gave her some water mixed with sugar” *(Father; 33 years old).

Most premature babies were reported to be very small and could not suck on their own. The mothers had to express their breast milk to feed their neonates using a cup or spoon. Although this is the best practice for the baby, the risk of infection remains.

## 4. Discussion

The majority of deaths occurred at home and was in the first three days of life. Care-seeking in the final illness was more common in children who were born at a health facility. Premature babies were more likely to die at home as compared to other neonates dying from other causes. The three leading causes of neonatal deaths in our study were prematurity, asphyxia, and infections. Birth asphyxia is increasingly accepted as being a poorly defined term, and “intrapartum-related deaths” has recently been proposed as an alternative. There is a spectrum of underlying causes [27–29]. Mortality is particularly high in the first day after birth and also the first three days of life which was again confirmed by this study. Most neonatal deaths are unrecorded in the health system as the majority die at home. Place and context of delivery is an important determinant for survival as it determines whether delivery is skilled or not. For example, in this study area, the most common first-line providers at birth are nurses/midwives, traditional birth attendants (TBAs), and relatives [12, 30]. Neonates delivered at health facilities were more likely to seek care from a health facility than those delivered at home. It is therefore possible that increasing facility births will improve care-seeking for neonatal illness. As expected, our study concurs with previous evidence that both births and deaths are largely unregistered as most of them occur out of reach of the health services [7] and very soon after birth [31].

Verbal autopsy has been widely used in ascertaining causes of death in children [15, 32, 33], but determining cause of death in neonates is particularly challenging given the nonspecific and overlapping clinical symptoms of several major causes of neonatal deaths [8, 32–34]. Likewise, the complexity of distinguishing birth asphyxia from other causes with use of the VA has been reported in other studies [8, 31, 35]. Methodological differences between studies make direct comparison difficulty [31]. There are two major limitations in our analyses. First, the information in this study was collected up to three years after the events had happened. The recall period could potentially affect the quality of detailed information collected for VA use. Baqui et al. utilized a five year recall period for children in Bangladesh and reported that increased recall times had no clear adverse effect on data quality [36]. Secondly, there was a high proportion (22%) of unresolved causes of neonate deaths, but this was similar to other studies [31]. The high number of unclassified deaths has affected the results of this study and represents a certain limitation. Although results from VA physicians' reviews are reported to vary considerably [37, 38], physicians' reviews provide more accurate results than the application of computerised algorithms [34, 39].

The adaptation of the VAs to the neonatal situation was a key element of our study. Despite the possible limitations of the VA approach as discussed in earlier studies [11, 20, 40], our study had provided new insights on care-seeking and causes of neonatal mortality. Setbacks for physicians' reviews include the high cost and the length of time needed for physicians to code the VA cause of death [40]. The strength of this study is the use of mixed methods approach by combining quantitative and qualitative data to look at various issues related to cause of death and care-seeking during the neonatal period. Quantitative and qualitative findings matched to a very large extent and provided important preliminary, first insights into the determinants that shape (i) the causes of death and (ii) care-seeking for the neonates in rural southern Tanzania.

 Understanding of local related factors related to early care-seeking for the neonate is important for improving neonatal health. In this study, we found that the most common reasons to delay care-seeking for the neonate outside home were (i) lack of funds, that is, cash and the time needed to be invested to go to the health facility including with cash mobilization, (ii) low accessibility due to distance to the referral health facility, (iii) preference of traditional healer for illness associated with spirits, and (iv) lack of knowledge to identify signs and symptoms of the illness related to the neonates. Other earlier studies have documented a similar patterns of delays in early recognition of illness, delay in transport, and access to appropriate care for serious maternal and newborn illnesses [41, 42]. Interestingly, a study from a rural setting in Indonesia showed that 22% of sick young infants were never seen by a care provider during the illness episode that led to death [43]. Families and communities' preferences for use of traditional remedies must be understood and addressed accordingly [42]. Delay in recognition of signs of neonatal illness by the caregiver was reported as a barrier to early care-seeking for the neonate and has also been reported elsewhere [44–46].

## 5. Conclusions

We present evidence on the cause-specific mortality and care-seeking for the neonates and show that the community perceives premature babies to be at greater risk of death. The majority of births are happening at home and assisted by unskilled attendants, where there is no possibility and approach to look after babies who are born premature. Combined with these findings, lack of appropriate knowledge to handle premature babies became also evident. Twin neonates were more commonly reported to be born premature and experienced more deaths compared to singletons. Efforts to promote early and exclusive breastfeeding must coherently address the importance of colostrums and the perception of a lack of milk in the first three days of life. Caregivers of neonates born in health facility were more likely to seek care for problems than caregivers of neonates born at home. Consequently, all efforts to increase awareness about early care-seeking for the neonates are important for improving newborn health. At the operational level, this will entail improving the antenatal care (ANC) counseling and the extension of services beyond facilities to the community to tackle the many constraints encountered by mothers and ANC providers [21, 47].

## Figures and Tables

**Table 1:** Care-seeking and causes of death.

	All (%)	Sought care during neonatal period		*P* value
		*n*	%	
Causes of neonatal death				
Prematurity	72 (33%)	25	35%	0.003
Asphyxia	49 (22%)	33	67%	
Infections	21 (10%)	11	52%	
Congenital abnormalities	10 (5%)	8	80%	
Other causes	19 (9%)	6	31%	
Unresolved	48 (22%)	20	41%	
**Total**	**219**	**103**		

Place of birth				
Home	125 (63%)	30	24%	0.0001
Health facility	74 (37%)	59	80%	
**Total**	**199**	**89**		
Missing data for 20 neonates				

Assistance during delivery				
Doctor	16 (7%)	14	88%	0.0001
Nurse/midwife	79 (36%)	57	72%	
Traditional birth attendants	75 (34%)	22	29%	
Other “relatives”	49 (22%)	10	20%	
**Total**	**219**	**103**		

Place of death				
Health facility	67 (31%)	61	91%	0.0001
Home	147 (69%)	40	27%	
**Total**	**214**	**101**		
Missing data for 5 neonates				

Gender				
Male	110 (52%)	47	43%	0.11
Female	103 (48%)	55	53%	
**Total**	**213**	**102**		
Missing data for 6 neonates				

Type of birth				
Singleton	185 (86%)	91	49%	0.09
Twins	31 (14%)	10	32%	
**Total**	**216**	**101**		
Missing data for 3 neonates				

**Table 2:** Perceived reasons for neonatal death.

Problem associated with assistance during labour and delivery	Unskilled attendants

	Lack of assistance at delivery
	Lack of capacity to handle complications

Problem associated with feeding practices	Majority of neonates are breastfed but some are given sweetened water, coconut juice, and tinned milk

Delay in care-seeking for the neonate outside the home	Lack of money
	The time taken to mobilize cash
	Distance to the health facility
	Lack of signs and symptoms of the illness

Premature birth	No mechanism to look after babies born premature after home birth
	Lack of knowledge on how to handle neonates born premature was common
	Use of cup or spoon to feed premature babies.
